# Arts on prescription intervention for primary healthcare patients with poor mental health or social isolation: a mixed-method study

**DOI:** 10.1186/s12875-025-02866-2

**Published:** 2025-05-10

**Authors:** Anita Jensen, Anders Halling, Mirnabi Pirouzifard, Martin Lindström

**Affiliations:** 1 Department of Clinical Sciences in Malmö, Lund University, and Center for Primary Healthcare Research (CPF), Lund University and Region Skåne, Malmö, Sweden; 2 Nord University, Levanger, Norway

**Keywords:** Arts on Prescription (AoP), Primary healthcare, Mental health wellbeing, Salutogenic health, Participatory arts activities, Social isolation, Loneliness, Social Prescribing (SP)

## Abstract

**Background:**

Primary healthcare providers are increasingly challenged in supporting patients with psychosocial needs. Arts on Prescription (AoP) has been shown to improve primary healthcare patients’ mental health wellbeing. The aim of the current study is to understand the psychosocial effect of participating in an Arts on Prescription programme.

**Methods:**

A total of 112 primary healthcare patients from 18 primary healthcare centres in Scania with mental health diagnoses depression and anxiety or social isolation participated in a 10-week group-based arts programme, twice a week for 2 h. A questionnaire with the Short Warwick Edinburgh Mental Wellbeing Scale (SWEMWBS), the Salutogenic Health Indicator Scale (SHIS) (for baseline and follow-up) and 14 sociodemographic and self-rated health covariates were collected as baseline. We also conducted 28 semi-structured interviews. We analysed data using paired t-test and a general linear regression model for change in SWEMWBS and SHIS. Qualitative data was analysed using a thematic approach.

**Results:**

The paired t-test showed highly significant results (p < 0.001) for increase in both SWEMWBS and SHIS. The general linear regression.

models show that women and participants with poorer self-rated health (SRH), more contacts with the healthcare system, other referrals from the primary healthcare centre, and no previous arts and culture engagement displayed significantly stronger associations with increase in SWEMWBS but not SHIS. Qualitative results highlight use of other interventions and difficulties navigating the health system.

**Conclusions:**

Our findings support a proportionate universalism (scale and intensity proportionate to the degree of need) approach indicating that AoP programmes could be valuable additions to healthcare pathways enhancing wellbeing for vulnerable populations. Findings should be interpreted with caution due to small sample size.

**Supplementary Information:**

The online version contains supplementary material available at 10.1186/s12875-025-02866-2.

## Introduction

The global mental health crisis is a pressing concern and labelled as the new pandemic by the World Health Organization (WHO). All over the world, mental health needs are at high levels, but responses are scarce and inadequate [1]. According to WHO, mental health disorders are projected to become the leading cause of disability by 2030 [2]. Primary healthcare providers are increasingly challenged in supporting the needs of their patients, especially those strongly affected by adverse social conditions [3]. Various illnesses and health conditions are significantly influenced by social determinants [4, 5], yet primary healthcare systems lack the necessary tools to address social issues and have limited established care pathways for patients experiencing mental health and social problems [6–8]. In addition, patients seek healthcare for non-clinical issues [9].

Support for Social Prescribing (SP) initiatives, such as Arts on Prescription, is growing among policymakers and governments. These initiatives are anticipated to help ease financial pressures on health services by tackling social determinants of health, reducing loneliness, and mitigating health inequalities [10–12]. A systematic review comprising 25 studies provides substantial evidence supporting the effectiveness of Arts on Prescription (AoP) in enhancing psychosocial wellbeing. The findings consistently demonstrate positive outcomes in terms of wellbeing, with participants frequently reporting that their engagement with AoP was meaningful. Additionally, AoP interventions were noted to foster social behaviour, which further contributed to improvements in both psychological and social wellbeing [13].

The aim of the current study was to investigate the effect of participation in a 10-weeks AoP programme for primary healthcare patients referred with stress, anxiety, mild to moderate depression, or those experiencing loneliness/social isolation to understand psychosocial effects of participating in the AoP programme. In this current article, we present quantitative and related qualitative findings, other parts of the study will be presented in other publications.

## Methods

### Recruitment and referral process

Primary healthcare patients were recruited from 18 different primary healthcare centres in Malmö, Sweden. After a consultation and assessment of suitability for the AoP programme by primary healthcare provider (GP, nurse, psychologist, rehabilitation coordinator etc.), patients were referred to the AoP programme coordinator.

### Inclusion criteria

Primary care patients, adults (18 years of age or above), mental disorders using the International Classification of Diseases 10 th Revision (ICD-10-SE): stress (F43.1, F43.2, F43.8, F43.8 A, F43.8 W, F43.9) anxiety (F40, F40.1, F41.0, F41.1, F41.2, F41.9) mild to moderate depression (F32.0, F32.0, F32.1, F33) (indexes for common mental health disorders (CMD) in primary healthcare or risk of loneliness/social isolation (ESI) – not in the diagnose index, created for the study protocol).

### Exclusion

Individuals under 18 years of age**,** individuals who did not speak or read Swedish (interpreter not available) or those with psychiatric conditions treated in specialized psychiatry (specialists in psychiatry).

### Ethics

The study received ethical approval from the Swedish Ethical Review Authority (Dnr 2021–02077). All participants were provided with detailed written and verbal information about the study and were informed of their right to withdraw at any time without any obligation to provide a reason. Informed consent was obtained from all participants for both their participation in the study and the publication of the findings. The research adhered to the ethical principles outlined in the Declaration of Helsinki for medical research. Participant anonymity was ensured throughout the study by pseudonymisation of data.

### Design of programme

The programme spanned 10 weeks, with participants engaging in activities twice weekly for two hours per session. To ensure continuity, these sessions were scheduled on the same weekdays throughout the programme's duration. A total of nine different arts and cultural institutions collaborated to design and facilitate the activities, offering a diverse range of artistic and cultural experiences. Notably, no therapists were involved in delivering the sessions. Table 1 provides an overview of the participating institutions, a brief description of the activities they offered, and the frequency of participant visits to each institution.

**Table 1:** The culture institutions and activities included in programme

Venue	Activity	Number of visits
Malmö Opera	Music activities: listening to mini concert flowed by discussion	1
Malmö Konsthall (gallery)	Guided tour of the exhibition and arts workshop	1
Malmö Stadsarkiv (city achieves)	Discovering new places and learning more about Malmö's history through a guided city walk	1
Malmö Live (music venue)	Introduction to the music venue, recording own song and trying instruments	3
Kollaborativet (preforming arts)	Interactive stage performance with focus a sensory experience	1
Malmö Bibliotek (library)	Shared reading method using short stories and poems	5
Malmö Muséer (museums)	Guided tours of the castle, the technical museum, and the collections as well as creative workshops	5
Form/Design Centre (arts and craft centre)	Guided tour of the current exhibition and arts workshop	1
Malmö Konstmuseum (arts museum)	City walk with a focus on public art and arts workshop	2

The programme was designed to offer participants a rich variety of aesthetic and creative experiences that engaged multiple senses—sight, hearing, and touch—while fostering involvement in practices centred on aesthetic appreciation. Activities included both active elements (such as creating, making, and participating) and receptive elements (such as listening, observing, and sensing), spanning diverse artistic disciplines such as literature, music, singing, visual arts, and performing arts. A total of 12 groups participated in the program, which ran from September 2021 to May 2024. The programme comprised 20 arts-based activities along with two additional meetings (an introductory session and a concluding session), totalling 24 sessions overall.

### Power calculation of Short Warwick Edinburgh Mental Wellbeing Scale (SWEMWBS)

In a pilot study conducted in 2020 with 24 individuals, the Mean (standard deviation, SD) SWEMWBS score before the programme was 15 (SD 5), and after the conclusion of the program it was 22 (SD 5) with a total of 25% drop outs. For the present study, we conducted the power calculations based on t-test (matched pairs) with mean of difference 5 and SD of difference 5 with total number of 10 groups (independent interventions with at least 7 individuals in respective groups), a power of 80%, significance level 5% and 25% expected drop outs to be able to show significant results. The size effect we attempted to detect was 5 points change in SWEMWBS between baseline and follow-up. In the study. The actual drop-out was 14% [14].

### Data collection

#### Quantitative data

The baseline data was collected in person at the information meetings and the follow-up data (10 weeks later) at the end meeting after completing the programme. Two different questionnaires were used: 1. Short Warwick Edinburgh Mental Wellbeing Scale SWEMWBS) (7 questions on wellbeing) and 2. Salutogenic Health Indicator Scale (SHIS) (12 questions on salutogenic indicators of health).

The SWEMWBS was used to measure mental well-being with the subitems asking participants how often they had been ‘feeling optimistic about the future; feeling useful; feeling relaxed; dealing with problems well; thinking clearly; feeling close to other people; able to make up their own mind about things’ over the past 2 weeks. Responses ranged from 1 (none of the time) to 5 (all of the time) on a 5-point Likert scale. Raw item-scores were summed and converted to metric total score using SWEMWBS conversion table. SWEMWBS score ranged from 7 (lowest possible mental well-being) to 35 (highest possible mental wellbeing) [15]. SWEMWBS has been validated in Sweden [16].

The SHIS scale includes a semantic differential consisting of 12 indicator items covering health-related dimensions: Tension, Illness, Energy experience, Energy level, Physical function, State of morale, Sleep, Expression of feelings, Concentration, Creativity, Resolution, Social capacity. A total index is calculated by totalling the value for all twelve questions. This means a maximum positive index value total of 72 and a corresponding minimum index value of 12 [17].

### Dependent variable

The outcome is the difference between the post-intervention score and the baseline score for SWEMWBS and SHIS, respectively, for each individual.

### Independent variables

The other 14 covariates were collected though background data and included: gender: women/men/other. Age: continuous variable 24–81 years. Civil status: living alone and married/cohabitating. Country of birth: born in Sweden or born abroad. Education level: upper secondary school (2-year secondary school or vocational school), 3- or 4- year secondary school, university education < 3 years, university 3 years or longer; other education; no education. Economic stress: being able to cover unexpected costs easily, yes or no. Work status: employed, student, pensioner, on sick-leave, parental leave/other leave, unemployed, homemaker. Self-rated health (SRH): very good, good, reasonable, poor, very poor. Chronic illness: diabetes, lung conditions, cardiovascular disease, hypertension. Medication for: chronic illness, mental health, other, no medication. Contact with healthcare system within the past year: 1–2 times, 2–5 times, 5–10 times, more than 10 times. Health locus of control (HLC): own effort has strong impact, only some impact, or no impact. The first alternative is defined as internal HLC and the other two are collapsed as external HLC. Other referrals within the healthcare system: no, yes- referrals to what? Arts engagement: visiting arts institutions/arts activities regularly yes or no.

### Qualitative data

A qualitative exploratory descriptive approach within an interpretive framework using one-to one semi-structured interviews explored the participants perspectives and experiences of the AoP programme. The interviews provided a description and detailed accounts of the participants'experiences and perspectives on a phenomenon [18]. Semi-structured interviews involve a set of open-ended questions that allow for spontaneous and in-depth responses [19]. An interview guide was used (see supplementary file 1). 28 semi-structured one-to one interviews were conducted. The first author conducted 26 interviews, and 2 interviews were conducted by a research assistant. The average length of the interviews was 38 min. The interviews were conducted between November 2021 and May 2024. 2–3 participants from each group volunteered to be interviewed. Interviews were conducted in Swedish, recorded, and transcribed verbatim. Themes identified in qualitative analysis are displayed in Table 2.

**Table 2:** Key themes, sub-themes, coding, and examples of quotes

Key theme	Sub-theme	Coding	Examples of quotes
*Background for referral*	Pharma	-Treatments -Pills -Diagnoses	“*I have been offered anti-depressants, sedatives and anxiety-reducing medication multiple times and I have tried them, but I always felt like… I didn’t really feel like they helped. So yes, I think this is more my way because I don’t want to medicate in that way, and I have tried, and it doesn’t work* (KuR 214)
	Healthcare activities	-Other referrals -Contact with healthcare system	*When one gets sick then one must also be very strong to get the right help…it’s so difficult*” (KuR158)

### Data analysis

Descriptive statistics were calculated in Table 3 as means and standard deviations (SD) for SWEMWBS, SHIS and age, and number of participants and prevalence (%) for the other variables. Continuous variables SWEMWBS, SHIS and age were tested for statistical significance (5% significance level) using Kruskal–Wallis test, and all other categorical variables were tested for statistical significance using Fisher exact test, in both cases because one of the three categories women, men and other only contains 3 participants (other) (Table 3).

**Table 3:** Descriptive characteristics for women (*n* = 83), men (n = 26) and other (n = 3). Means and standard deviations (SD) for SWEMWBS, SHIS and age. Number of participants and prevalence (%) for the other variables. *N* = 112

	**All**	**Gender**			
		**Women**	**Men**	**Other**	^***‡***^***P-value***
		83 (74.1)	26 (23.2)	3 (2.7)	
**SWEMWBS**					
Baseline, mean (SD)	17.7 (5.0)	18.0 (4.8)	17.0 (5.9)	15.0 (1.7)	0.302^£^
Follow-up, mean (SD)	22.5 (5.1)	23.5 (4.8)	19.7 (4.6)	19.7 (7.2)	**0.002**^£^
**DIFF SWEMWBS**, mean(SD)	4.8 (5.2)	5.5 (5.1)	2.7 (5.4)	4.7 (5.5)	**0.036**^£^
**SHIS**					0.087^£^
**Baseline**, mean (SD)	30.7 (8.8)	31.0 (8.3)	31.0 (10.3)	20.3 (3.5)	0.092^£^
**Follow-up**, mean (SD)	43.6 (10.6)	44.9 (10.0)	40.3 (11.7)	37.3 (11.9)	**0.011**^£^
**DIFF SHIS**, mean(SD)	12.9 (10.8)	13.8 (10.1)	9.3 (12.6)	17.0 (8.7)	
**Age**, mean (SD)	59.0 (13.9)	57.7 (14.0)	63.8 (10.6)	53.7 (10.6)	0.088^£^
**Depression**	34 (30.4)	22 (26.5)	11 (42.3)	1 (33.3)	0.229^***&***^
**Anxiety**	37 (33.0)	32 (38.5)	4 (15.4)	1 (33.3)	0.070^***&***^
**Stress**	24 (21.4)	19 (22.9)	4 (15.4)	1 (33.3)	0.484^***&***^
**Social isolation**	17 (15.2)	10 (12.0)	7 (26.9)	0 (0.0)	0.168^***&***^
**Living alone (vs. married/cohabitating)**	77 (68.7)	52 (62.7)	22 (84.6)	3 (100.0)	0.075^***&***^
**Country of birth – born outside Sweden**	29 (25.9)	25 (30.1)	3 (11.5)	1 (33.3)	0.121^***&***^
**Education**					
Primary education	13 (11.6)	9 (10.8)	4 (15.4)	0 (0.0)	0.358^***&***^
Secondary education	54 (48.2)	42 (50.6)	12 (46.1)	0 (0.0)	
Tertiary education	45 (40.2)	32 (38.6)	10 (38.5)	3 (100.0)	
**Economic stress** (yes)	79 (70.5)	62 (74.7)	15 (57.7)	2 (66.7)	0.195^***&***^
**Self-rated health**					
Very good/good	8 (7.1)	6 (7.2)	2 (7.7)	0 (0.0)	0.441^***&***^
Reasonable	55 (49.1)	43 (51.8)	12 (46.1)	0 (0.0)	
Very poor/poor	49 (43.8)	34 (41.0)	12 (46.1)	3 (100.0)	
**Diabetes**	18 (16.1)	7 (8.4)	11 (42.3)	0 (0.0)	** < 0.001**^***&***^
**Lung disease**	11 (9.8)	8 (9.6)	2 (7.7)	1 (3.3)	0.362^***&***^
**Cardiovascular diseases**	14 (12.5)	8 (9.6)	5 (19.2)	1 (33.3)	0.152^***&***^
**Hypertension**	34 (30.4)	26 (31.3)	8 (30.8)	0 (0.0)	0.761^***&***^
**Mental health medication**	50 (44.6)	35 (42.2)	13 (50.0)	2 (66.7)	0.573^***&***^
**Other medicine**	82 (73.2)	61 (73.5)	20 (76.9)	1 (33.3)	0.282^***&***^
**No medicine**	9 (8.0)	7 (8.4)	1 (3.9)	1 (3.3)	0.183^***&***^
**Contact with health system**					
1–5 times/year	37 (33.0)	24(28.9)	13 (50.0)	0 (0.0)	0.078^***&***^
6- times/year	75 (67.0)	59 (71.1)	13 (50.0)	3 (100.0)	
**Health locus of control**					
Internal	82 (73.2)	64 (77.1)	16 (61.5)	2 (66.7)	0.224^***&***^
External	30 (26.8)	19 (22.9)	10 (38.5)	1 (33.3)	
**Other referrals** (None)	57 (50.9)	39 (47.0)	17 (65.4)	1 (33.3)	0.228^***&***^
**Arts engagement** (None)	76 (67.9)	58 (69.9)	16 (61.5)	2 (66.7)	0.763^***&***^

^£^ *p*-value: Kruskal–Wallis Test

^&^ *p*-value: Fisher exact test

^*‡*^ Bold *p* < *0.05*

**Fig. 1:**
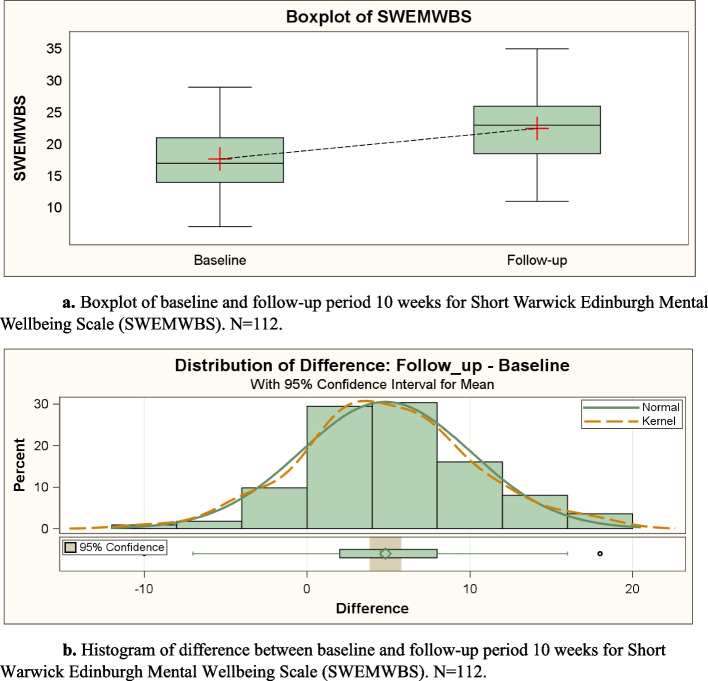
a. Boxplot of baseline and follow-up period 10 weeks for Short Warwick Edinburgh Mental Wellbeing Scale (SWEMWBS). *N* = 112. **b**. Histogram of difference between baseline and follow-up period 10 weeks for Short Warwick Edinburgh Mental Wellbeing Scale (SWEMWBS). *N* = 112

**Fig. 2:**
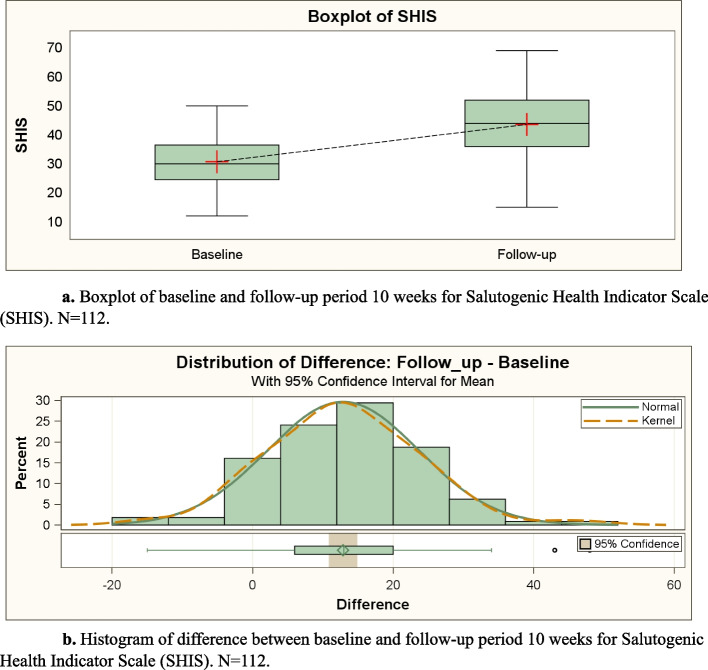
a. Boxplot of baseline and follow-up period 10 weeks for Salutogenic Health Indicator Scale (SHIS). *N* = 112. **b**. Histogram of difference between baseline and follow-up period 10 weeks for Salutogenic Health Indicator Scale (SHIS). *N* = 112

Boxplots and histograms of distributions of change in SEMWBS and SHIS between baseline and follow-up were performed in order to assess magnitude and distributions of change among the 112 participants in order to use paired t-test (see Figs. 1, 2, and Tables 4, 5).

**Table 4:** Mean, standard deviation (SD), median, minimum, and maximum for baseline, follow-up and difference between baseline and follow-up (DIFF) with paired t-test for DIFF for SWEMWBS. *N* = 112

**Variable**	Minimum	Maximum	Mean	SD	Median	*p-value*
DIFF	−10	18	5	5	5	< 0.001
Follow-up	11	35	22	5	23	
Baseline	7	29	18	5	17

**Table 5:** Mean, standard deviation (SD), median, minimum and maximum for baseline, follow-up and difference between baseline and follow-up (DIFF) with paired t-test for DIFF for SHIS. *N* = 112

**Variable**	Minimum	Maximum	Mean	SD	Median	*p-value*
DIFF	−15	48	13	11	13	< 0.001
Follow-up	15	69	44	11	44	
Baseline	12	50	31	9	30	

Data analysis was conducted using a paired t-test to determine if there was a significant difference in the means of the baseline and follow-up data by assessing the difference in means between these paired observations (baseline and follow-up) for SWEMWBS and SHIS, respectively (see Figs. 1, 2 and Tables 4, 5).

The paired t-test was used to assess significant differences (5% significance level) between the SWEMWBS item at baseline and the SWEMWBS item at follow-up after10 weeks, and paired t-testwas also calculated for the SHIS item at baseline compared with the SHIS item at follow-up after 10 weeks (see Figs. 1, 2, and Tables 4, 5). The preconditions for use of parametric t-test were tested with histogram and Q-Q-plot, Shapiro Wilks test, control of skewness and kurtosis, and descriptive statistics including control of symmetric distribution comparing median and mean (which should be equal). The results of these tests supported the application of parametric tests and statistical methods.

General linear regression models were calculated to assess associations between differences in SWEMWBS at baseline compared to follow-up, and the other variables (See Table 6). These variables include gender, age, inclusion diagnosis and social isolation, civil status, country of birth (born in Sweden or abroad), economic stress, self-rated health, somatic diagnoses (diabetes, lung disease, cardiovascular disease and/or hypertension), medication, contact with the healthcare system, health locus of control, other referrals from the primary healthcare centre and regular participation in cultural activities (yes/no). In model 1, all variables were included to assess statistically significant associations (5% significance level) with change in SWEMWBS at baseline compared to follow-up. In model 2, all variables that were not significantly associated with change in SWEMWBS were excluded (Table 6). The general linear regression models were also calculated to assess associations between differences in SHIS at baseline compared to follow-up and the same variables listed above. In model 1, all variables were included. In model 2, all variables that were not significantly associated with change in SHIS between baseline and follow-up were excluded (Table 7). In the general linear regression model 1 (mean level + random variation), normality assumptions were tested on the outcome variable by Shapiro-Wilks W test. Furthermore, Skewness and Kurtosis were in accordance with a common normal distribution was tested. In model 2, the same checks were conducted on the residuals from the model to verify normality on the random errors. In Tables 6 and 7 only 109 of the 112 participants were included in the models because the category “other” than women or men was too small to be included in the multiple general linear regression models. In all other calculations all 112 participants were included, because none of the items (variables) included in our multiple linear regression analyses had any missing values (no missing values). The SAS software version 9.4 was used in all analyses.

**Table 6:** General linear model (GLM) predicts the difference between baseline and follow-up for SWEMWBS for women (*n* = 83), men (*n* = 26). *N* = 109

		Model 1		Model 2	
**Explanatory variables**	Ref	β	*P-value*^***‡***^	Est	*P-value*^***‡***^
**Gender**	Women	−3.04	**0.024**	−2.48	**0.029**
**Age**		−0.07	0.168		
**Inclusion diagnosis**					
Depression	Social isolation	1.43	0.382		
Anxiety		−0.90	0.587		
Stress		−1.91	0.274		
**Living alone**	Married/cohab	0.68	0.573		
**Country of birth**	Sweden	−1.12	0.344		
**Education**					
Primary	Tertiary	−2.81	0.123		
Secondary		0.48	0.661		
**Economic stress**	No	−0.51	0.703		
**Self-rated health**					
Very good/good	Reasonable	1.16	0.590	0.15	0.937
Very poor/poor		2.23	**0.039**	2.44	**0.016**
**Diabetes**	No	0.38	0.806		
**Lung disease**	No	−0.42	0.815		
**Cardiovascular disease**	No	1.40	0.411		
**Hypertension**	No	0.55	0.664		
**Mental health medication**	No	−0.80	0.522		
**Other medicine**	No	−2.12	0.187		
**No medicine**	Any med	−4.86	0.054		
**Contacts with the healthcare system**	Less contact	2.25	**0.049**	2.02	0.054
**External health locus of control**	Internal	−0.61	0.619		
**Other referrals from healthcare center**	No	2.14	**0.049**	1.20	0.204
**Arts and culture engagement**	Yes	2.88	**0.012**	2.89	**0.005**

Model 1: All variables included

Model 2: Only significant variables from model 1 included

^*‡*^Bold *p* < *0.05*

**Table 7:** General linear model (GLM) predicts the difference between baseline and follow-up for SHIS for women (*n* = 83), men (*n* = 26). *N* = 109

		Model 1		Model 2	
**Explanatory variables**	Ref	β	*P-value*^***‡***^	Est	*P-value*^***‡***^
**Gender**	Women	−5.91	**0.047**	−4.50	0.064
**Age**		−0.17	0.099		
**Inclusion diagnosis**					
Depression	Social isolation	2.82	0.437		
Anxiety		−3.30	0.369		
Stress		−2.69	0.486		
**Living alone**	Married/cohab	0.99	0.710		
**Country birth**	Sweden	−1.07	0.683		
**Education**					
Primary	Tertiary	0.64	0.874		
Secondary		1.44	0.551		
**Economic stress**	No	−1.57	0.597		
**Health status**					
Very good/good	Reasonable	2.35	0.623		
Very poor/poor		1.86	0.433		
**Diabetes**	No	−0.43	0.900		
**Lung disease**	No	−2.54	0.522		
**Cardiovascular disease**	No	4.47	0.238		
**Hypertension**	No	−1.25	0.657		
**Mental health medication**	No	−0.80	0.772		
**Other medicine**	No	1.18	0.740		
**No medicine**	Any med	−6.19	0.264		
**Contact with healthcare system**	Less contact	3.87	0.125		
**External health locus of control**	Internal	2.72	0.319		
**Other referrals from healthcare center**	No	4.33	0.073		
**Arts and culture engagement**	Yes	3.07	0.219		

Model 1: All variables included

Model 2: Only significant variables from model 1 included

^*‡*^ Bold *p* < *0.05*

The qualitative data was analysed using a thematic approach [20](Braun & Clark, 2018) where we systematically categorizing and labelled data to identify patterns, themes, and relationships.

The process analysing the qualitative data involved six distinct phases, namely: 1. *Familiarization with data*; 2. *Generating initial code*; 3. *Searching for themes*; 4. *Involved reviewing themes;* 5. *Defining and naming themes*; 6. *Producing the report.* Five themes were identified. In this article, we consider the theme *Background to referral* together with the quantitative results (see Table 2). The analysis was conducted by two researchers, including the first author.

## Results

### Background to referrals

All patients referred from primary healthcare centres were diagnosed with a mental health disorder or were socially isolated/lonely (see methods sections for inclusion criteria). Struggling with various difficulties due to mental health disorders, a patient expressed how daily tasks were challenging because of stress: *“My nerve system is triggered very easily and it…it’s hard to constantly try to deal with those stress reactions”* (KuR112).

Some have been on sick leave for a long time, others not, some battled with other illness and issues (including chronic pains, lung and cardiovascular disease, hypertension, exhausting disorder or experiences of trauma and bereavement) as well as mental health disorders (depression, stress, anxiety) or transitions in life (e.g. moving, retiring, made redundant). A patient describes how the referral to AoP was made while visiting the GP for receipt renewal: *“So I wanted new sleeping pills. I spoke to the doctor and…he asked questions about my situation and…that’s when this [AoP]came up. So that is how is started”* (KuR179). Another patient describes a journey through taking different pharmaceutical treatments without experiencing any health improvement as well as a desire for finding other non-pharmacological solutions: “*I have been offered anti-depressants, sedatives and anxiety-reducing medication multiple times and I have tried them, but I always felt like… I didn’t really feel like they helped. So yes, I think this is more my way because I don’t want to medicate in that way, and I have tried, and it doesn’t work* (KuR 214). In addition, a patient expresses it as fight about medication before ending up with another diagnosis *“I had a real struggle with my doctor who forced antidepressant on me when actually…they gave me an exhaustion diagnosis”* and some experienced waiting a long time for treatment “*There was a lack of psychologist so there was a very long wait and so on and I ended up on a waiting list for CBT and it took a very long time”* (KuR161).

In this way, some patients had experiences challenges getting the right care, diagnose and having to wait for treatment.

### Descriptive characteristics

Descriptive characteristics are presented in Table 3 which include covariates such as:

### Participant profiles

112 participants completed the programme (baseline- follow-up) and 16 dropped out (various reasons: moving away, gaining employment, too ill, job training, other commitments etc.).

### Gender identity and age

Women *n* = 83; Men *n* = 26; Other *n* = 3. Age: Median age: 61 years of age, Average age: 59 years of age, youngest participant was 25 years old and oldest 81 years of age.

### Country of origin

The proportion born outside Sweden was 26%. Countries of birth were Ukraine, Poland, Serbia, Romania, Hungary, Colombia, Finland, Mexico, former Yugoslavia, Kurdistan (region), Iran, Iraq, Lebanon, Denmark, Germany, Eritrea, Bolivia.

### Reason for referral

33.0% had anxiety related disorders; 30.4% had a depression diagnosis; 21.4% had a stress diagnosis; 15.2% were lonely or/and socially isolated.

### Self-reported chronic diseases

48% of participants reported one or more of the following chronic diseases: diabetes, lung disease, cardiovascular disease, and hypertension in addition to the referral diagnosis.

### Health locus of control (HLC)

73,2% of the participants reported that regarded their own contribution to remain in good health as very important (internal HLC), while 26,8% reported that own contribution could some importance (external HLC).

### Contact with the healthcare system

The average participant had had contact with the health care system between 6 and 10 times in the past year.

### Self-rated health (SRH)

Regarding SRH, the participants rated themselves as follows: Good: 7,1% (*n* = 8), Reasonable = 49,1% (*n* = 50), Poor/very poor = 43,8% (*n* = 49). For comparison, the random sample of the general population has regularly reported approximately 70% good/very good and approximately 30% reasonable/poor/very poor in the public health surveys/questionnaires conducted by Region Skåne in 2000, 2004, 2008, 2012, 2019 and 2022 [21].

### Other interventions

49,1% of the patients reported that they had previously been referred to and participated in other interventions attempting to manage depression, stress and anxiety through different activities offered through primary healthcare (e.g., mindfulness, mediyoga (medical yoga), nature rehabilitation (NuR), basic body awareness, acupuncture, cognitive behavioural therapy (CBT)) as well as taking anti-depression medication. Some have found navigating the health system challenging, as a patient expressed:” *When one gets sick then one must also be very strong to get the right help…it’s so difficult*” (KuR158) and was disillusioned with the support offered.

The patients described their experiences with healthcare interactions, with several emphasizing the ongoing challenges and efforts to find effective treatment approaches. One patient, for example, expressed difficulty in engaging with mindfulness practices: *"I couldn't get my head around this idea of going into yourself and…it didn't work"* (KuR147). Some patients reported that they had benefitted from interventions such a yoga: *“I really liked the mediyoga…I still do that privately at home”.* Another patient expressed positive benefits from being referred to NuR after finding a foothold in the activity “…*two years ago, NuR on farm…it took a while for me to get into it and to like land and relax”* (KuR112).

### Questionnaires

Using two questionnaires (SWEMWBS and SHIS), data was registered at baseline and at follow-up 10 weeks later. Figure 1 shows that individual-level differences in SWEMWBS between follow-up and baseline were approximately normally distributed, a finding that made parametric testing with t-test possible. Table 4 shows that means and medians were almost equal, and the paired t-test was highly significant (p < 0.001). Figure 2 shows that individual-level differences in SHIS between follow-up and baseline were also approximately normally distributed, so parametric testing with paired t-test was possible also for change between follow-up and baseline in SHIS. Table 5 shows that the paired t-test was highly significant also for change in SHIS (p < 0.001) (see Tables 4 and 5).

In the general linear regression model (see Table 6) change in SWEMWBS between baseline and follow-up was significantly associated with female gender, β = −3.04 (*p* = 0.024), very poor/poor self-rated health, β = 2.23 (*p* = 0.039), more contact with the healthcare system, β = 2.25 (*p* = 0.049), other referrals from the primary healthcare centre, β = 2.14 (*p* = 0.049), and no participation in cultural activities, β = 2.88 (*p* = 0.012). In Table 7, the results in model 1 show that change in SHIS between baseline and follow-up was only significantly associated with gender, β = −5.91 (*p* = 0.047), which indicated a significantly stronger effect of the intervention on women. No significant associations were for observed for the other variables.

## Discussion

Our main findings showed a significant increase in mental health wellbeing as effects of participation in an AoP programme (see Tables 4, 5).

### More benefit for women

A major finding of the multiple linear regression models is that women and participants with poorer SRH, who had had more contacts with the healthcare system in the past year, had other referrals from the primary healthcare centre and with no previous arts and culture engagement displayed significantassociations with increase in SWEMWBS but not SHIS (see Tables 6, 7). Another study shows that beyond promoting wellbeing an AoP programme was particularly effective in the promotion of wellbeing and in targeting women, older people, and people from lower socioeconomic groups [22], which supports the results of this study.

The results of the multiple general linear regression models of changes in SWEMWBS and SHIS, respectively, show that effects of the intervention were stronger among women. Change in SWEMWBS was significantly associated with gender (greater effect of change in SWEMWBS among women), poorer SRH, more contact with the healthcare system, other referrals from the primary healthcare centre, and other cultural participation. In contrast, change in SHIS showed significant association with gender but no other variables. Given the high and well-documented validity of the SWEMWBS instrument, this indicates that women and the most isolated and vulnerable benefitted most from the intervention.

Interventions aimed to reduce social and socioeconomic health differences should address all levels of society and not exclusively groups that are most vulnerable, however they should also consider particularly vulnerable population groups. Proportionate universalism may be an efficient strategy. Proportionate universalism means that while interventions are universal and directed towards the entire population, e.g. encouraging cultural engagement, interventions specifically targeted towards limited population groups with special needs may be launched to improve health and wellbeing for such population groups [23, 24]. Patient groups experiencing poor mental health and social isolation who are in contact with primary healthcare centres are an example of such a restricted population group with special needs. Other AoP studies have showed positive health outcomes for primary healthcare patients with poor health [13, 25, 26].

The majority of the participants in the study were women (*n* = 83). In other AoP studies, authors have reflected on the gender distribution among participants and concluded that women are more likely to join community-based social groups than men, and this may explain the preponderance of women [13]. In this study, it is not possible to determine whether an inherent bias exists due to more women than men being offered the opportunity to participate in the programmes through their primary healthcare or if the creative activities appeal more to women. Research suggests that women are more likely to engage in the arts [27].

### Navigating the health system

The qualitative data revealed that patients within the study population encountered considerable challenges in obtaining appropriate care, receiving accurate diagnoses, and accessing timely treatment. Some participants reported to have experienced long waiting periods for treatment, and several had been referred to various mental health interventions with mixed results. These findings highlight the complexities surrounding mental health care in primary settings, where treating mental health conditions remains a significant challenge [28]. Integrating mental health services within primary care structures has proved difficult [29]. Nevertheless, failing to adequately address mental health disorders not only impacts individual wellbeing but also imposes substantial financial costs on healthcare systems and society as a whole [30]. Addressing these systemic barriers to mental health care could improve outcomes and reduce the long-term financial burden of untreated mental health conditions.

In addition, based on reported challenges in getting appropriate support and the findings that women and vulnerable patients benefit the most suggest that an AoP intervention could be beneficial for group of patients that confronts both psychosocial and systemic difficulties. Such findings are also reported in another AoP studies [13, 31].

### Self-rated health

SRH is considered a key measure in epidemiological studies and is an effective predictor for mortality and morbidity [32]. Compared with the general population [21], the SRH reports from this study show a much lower level of SRH with the majority of the participants in the study viewing their health status to be reasonable to poor. Only 7–8% of participants indicated very good or good self-rated health in the questionnaire at baseline, in fact none indicated very good self-rated health. These results stand in sharp contrast to the self-reporting by random samples of the general population aged 18–80 (84) years old in the public health surveys regularly conducted in Scania every fourth year. These surveys show that approximately 70% of the general population in Scania regard their health as very good or good. Our study population is apparently a very vulnerable section of the general population and likely to have increased mortality and morbidity rates. In addition, 48% of participants had one or more of the following chronic diseases: diabetes, lung disease, cardiovascular disease, or hypertension. This may also be due to the relatively high median age (61 years of age). Many participants had also had contact with the healthcare system between 6 and 10 times in the past year, which suggests that they have critical health needs.

### Protective health and wellbeing factors

The evidence associated with a life course approach suggests that positive and negative factors for wellbeing accumulate throughout life and a policy response that seeks to maximize protective factors while minimizing risks can be successful in achieving wellbeing and health gains [4, 5]. The results of this study demonstrate a significant improvement in wellbeing and salutogenic health, suggesting that incorporating AoP programmes into health policies may enhance protective factors, including psychological wellbeing, positive emotions, and coping skills. Furthermore, AoP programmes foster positive social factors, such as a sense of community belonging [33, 34]. Collectively, these elements may enhance individuals'capacity to access internal resources, ultimately contributing to a reduction in negative mental health outcomes.

### Loneliness and social isolation

15% of the study population were referred due to loneliness or social isolation (accessed by healthcare provider) and the average person in this group was over 65 years old, indicating that the loneliness/social isolation was predominantly present in the older population. Loneliness and social isolation are considered non-clinical needs but risk factors that can lead to increased risk for early mortality [35, 36]. For this reason, it is pivotal that primary care providers can offer pathways to reconnect this group with social communities. Findings from other AoP studies have also highlighted decrease in loneliness among participants ( [37, 38].

### Strengths and limitations

Strengths include the longitudinal study design with baseline and follow-up reporting. A longitudinal study design enables researchers to investigate how outcomes evolve, which may suggest possible effects of interventions. A sample of patients with well-defined diagnoses of poor mental health or patients that were assessed as socially isolated was selected. The use of a mixed methods methodology approach may be regarded as a strength. A mixed method study enables detailed, contextualized insights of qualitative data and the generalizable, externally valid insights of quantitative data. In this study, the qualitative data provides us with insight of how the patients feel about their interaction with the health system, health situations, other referrals and the care provided. The use of the validated and internationally well-documented SWEMWBS instrument is a strength [15, 39]. The fact that magnitude of change in the valid SWEMWBS instrument between baseline and follow-up shows significant positive associations with poor self-rated health, more contacts with the healthcare system, other referrals from healthcare centre and no previous arts and culture engagement further strengthens the argument for specifically directed interventions based on the principle of proportionate universalism.

Limitations include a comparatively small study sample, although statistical power calculations were conducted prior to the intervention. Another limitation was the lack of control group. Another similar study used a control group with a follow-up and reported statistical significance for decrease in anxiety and depression in the intervention group [25]. An optimal control group would have contained a group of patients with the same diagnoses and social problems (isolation) as the treatment group receiving the intervention. We recommend further studies to include a control group, more specific measuring tools for different mental health diagnoses (i.e. anxiety, depression, stress) as well as longitudinal follow-ups.

## Conclusion

In conclusion, this study shows that participating in an AoP programme significantly improves mental health and wellbeing, especially among women, individuals with poorer SRH, and those frequently engaged with the healthcare system. The AoP programme fostered psychological wellbeing, positive emotions, coping skills and promoted social connections with notable benefits for socially isolated and vulnerable participants and older adults experiencing loneliness.

Study limitations include a small sample size and lack of a control group that suggests the need for further research. Overall, the findings support a proportionate universalism approach, indicating that AoP programmes could be valuable additions to health policy enhancing wellbeing for vulnerable populations. Given the challenges of treating mental health in primary healthcare, AoP programmes could be offered as an alternative care pathway for patients with poor mental health wellbeing standing along or alongside other treatment. Implementation could be enhanced through social prescribing models and cross-sector collaborations that integrate primary healthcare, community services, and the arts.

## Supplementary Information

Supplementary Material 1

## Data Availability

The datasets generated and/or analysed during the current study are not publicly available but are available from the corresponding author on reasonable request.
